# Which Digital-Output MEMS Magnetometer Meets the Requirements of Modern Road Traffic Survey?

**DOI:** 10.3390/s21010266

**Published:** 2021-01-03

**Authors:** Michal Hodoň, Ondrej Karpiš, Peter Ševčík, Andrea Kociánová

**Affiliations:** 1 Department of Technical Cybernetics, Faculty of Management Science and Informatics, University of Žilina, 010 26 Žilina, Slovakia; karpis@pd.uniza.sk (O.K.); peter.sevcik@fri.uniza.sk (P.Š.); 2 Department of Highway Engineering, Faculty of Civil Engineering, University of Žilina, 01026 Žilina, Slovakia; andrea.kocianova@uniza.sk

**Keywords:** magnetometer, traffic, vehicle, classification, measurement, detection

## Abstract

Present systems for road traffic surveillance largely utilize MEMS magnetometers for the purpose of vehicle detection and classification. Magnetoresistive sensing or LR oscillation circuitry are technologies providing the sensors with the competitive advantage which lies in the energy efficiency and low price. There are several chip suppliers on the market who specialize in the development of these sensors. The aim of this paper is to compare available sensors from the viewpoint of their suitability for traffic measurements. A summary of the achieved results is given in the form of the score for each sensor. The introduced sensor chart should provide the audience with knowledge about pros and cons of sensors, especially if intended for the purposes of road traffic surveillance. The authors in this research focused on the specific situation of road traffic monitoring with magnetometers placed at the roadside.

## 1. Introduction

At present, non-intrusive automatic traffic counters are mainly used for short-term road traffic surveys. The counters are located mostly next to the road (e.g., cameras and microwave radars), across the road (pneumatic counters), or directly on the road surface in the middle of the traffic lane (magnetic traffic counters). In recent years, research and development have focused on counters based on magnetometers, which are already commonly used as a replacement for induction loops built into the road pavement. The possibility to use magnetometers placed next to the road is verified, which is very advantageous from the point of view of short-term surveys. However, it brings several challenges that need to be solved. One of the important ones is that in such a location the response from passing vehicles is much lower than when the sensor is located directly in the middle of the lane. Moreover, the amplitude of the record decreases significantly as the distance increases. Therefore, it is necessary to choose a suitable magnetometer that will be sufficiently sensitive and reliable, especially under real traffic conditions.

Currently, magnetometers based on micro-electro-mechanical systems (MEMS) technology are mainly used for vehicle detection. The situation on the MEMS market is evolving and several sensors (e.g., the HMC5983, Honeywell, Charlotte, NC, USA and the LSM303DLHC, STMicroelectronics, Geneva, Switzerland), which we have used in the past [1], are no longer available. Our intention is to compare the currently available magnetometers in terms of their suitability for vehicle detection if the sensor is located next to the road. Recently we compared 10 different sensors in the laboratory [2,3] from which we selected the four best. Now we are mainly interested in realistically achievable vehicle detection results.

Most vehicles are still based on internal combustion engines, which do not generate a significant magnetic field of their own. Detection of vehicles by magnetometers is based on the effect of ferromagnetic materials contained in vehicles on the Earth’s magnetic field. As the magnitude of the Earth’s magnetic field is approximately 50 μT, it is necessary to focus on sensors with the highest possible sensitivity. Sensitivity is usually expressed in terms of the number of quantization steps per μT (LSB/μT). 

The paper is organized as follows: Section 2 is focused on the state-of-the-art in the area of magnetometers used for traffic survey. In Section 3, basic parameters of selected sensors are presented. Section 4 contains a description of hardware used in all experiments. Section 5 is devoted to the laboratory experiments. In Section 6, the results of real-world measurements are presented and discussed. Detection algorithm and results are described in Section 7. Section 8 summarizes results of the sensor evaluation. Finally, Section 9 contains brief conclusions and a description of future research plans.

## 2. Related Work

The intelligent transportation systems (ITS) of the 21st century demand reliable, real-time vehicle volume and classification information. Different systems for different purposes integrate digital anisotropic magnetometers in their designs, as for example in parking lots [4], for stop detection at signalized intersections [5], for real-time traffic surveillance [6], etc. However, most magnetometer sensors applied in the field of traffic monitoring and classification are low-cost and energy-efficient substitutes of other technologies, such as for example inductive loops or cameras. Scenarios of traffic monitoring and vehicle classification were also the intended application areas of the sensors investigated throughout the paper. An extensive body of research is still carried out in this field, whilst findings in following articles were considered during our measurements.

The authors in [7] focused on the utilization of magnetometer sensors for length-based vehicle classification, where through their computationally efficient classification model reached classification accuracy of 97.70% by using of the anisotropic magnetoresistance (AMR) effect. 

In [8], the authors through the usage of a HMC1502 AMR magnetometer (Honeywell) presented an efficient approach to the modeling and classification of vehicles using their magnetic signatures. They proposed a sensor-dependent approach for modeling the obtained magnetic signature of each vehicle. The orientation of the used magnetometer was towards the Y and Z-axis components of the magnetic field.

The utilization of cost-effective solution for on-road traffic monitoring based on the usage of RM3100 magnetometers (PNI Sensor Corporation, Santa Rosa, CA, USA), was introduced in [9]. The authors, through modelling of the local magnetic field perturbations caused by moving vehicles, extracted the magnetic waveform characteristics for vehicle identification and speed estimation. They equipped their system with wireless connectivity to broadcast the information about vehicle types, volume and speeds. 

The authors in [10] also used a cheap AMR magnetometer, installed roadside, for road vehicle recognition and classification. Their system was used for measuring magnetic field changes for the detection of passing vehicles, and for recognition of vehicle types. Mel frequency cepstral coefficients were introduced to analyze vehicle magnetic signals and extract them as vehicle features with the representation of cepstrum, frame energy, and gap cepstrum of magnetic signals. According to this analysis they presented a special algorithm for the classification of the magnetic features of four typical types of vehicles—sedans, vans, trucks and buses. 

The authors in [11] proved the advantages of using of AMR magnetometers over Hall sensors, giant magnetoresistance (GMR) sensors, and tunneling magnetoresistance (TMR) sensors as a low cost as well as low power solution for vehicle detection sensing. In their research, they used two AMR sensor nodes spaced 1 m apart based on HMC5883L magnetometers (Honeywell) to measure vehicle speeds on the road. According to the sampling frequency limit their system could measure vehicle speeds up to 100 km/h. 

Research in [12] described the possibility of adding another sensor to magnetometers for vehicle tracking on roadways to build a low-cost, low-complexity vehicle tracking sensor platform for highway traffic monitoring. Their approach was based on multirate particle filtering that fused the measurements from two different sensors. The sensors were an accelerometer and a magnetometer, and they operated with different sampling rates. 

The authors in [13] utilized a FXOS8700 magnetometer as the basis of the presented smart wireless sensor for traffic monitoring with efficient and reliable algorithms for vehicle detection, speed and length estimation and classification. They introduced this AMR sensor as a suitable alternative for inductive loop sensors. Their system was portable, reliable, and cost-effective with an affordable price of 50 USD. The authors claimed 99.98% detection accuracy, 97.11% speed estimation accuracy, and 97% length-based vehicle classification accuracy. 

Detection of vehicles based on HMC5883L magnetometers was presented in [14]. The sensors were placed roadside and measured traffic in the adjacent lane. The authors proposed adapting a threshold state machine algorithm to detect vehicles. Their sensor system is wireless, compact, and cost-effective while achieving high accuracy and viability in urban environments. 

A comprehensive analysis of traffic surveillance based on magnetic technology and the corresponding wireless sensor networks, pointing out vehicle detection and counting, speed estimation and vehicle classification applications was presented in [15]. The authors developed a low-cost and energy-efficient type of multi-function wireless traffic magnetic sensor for vehicle detection at the jam flow conditions. Honeywell HMC1001 and HMC1002 magnetic sensors were used for this purpose.

The advantages of magnetic detector-based sensors compared to other technologies were also presented in [16]. The authors there presented a single magnetic detector system based on a Honeywell HMC5843 magnetometer. They presented a technique for false detection filtering where the vehicle classes are estimated using a feedforward neural network which was implemented in the detector control unit. For the training of the neural network the back-propagation algorithm was used with different training parameters.

A three axis digital HMR2300 AMR magnetometer (Honeywell) was used for the road vehicle classification in [17]. The vehicles were classified into four groups—hatchbacks, sedans, buses, and (MPVs). Authors developed classification algorithm based on analysis of time domain and frequency domain features in combination with three common classification algorithms in pattern recognition.

Magneto-inductive RM3100 sensors were used in [18] for road vehicles velocity estimation. The developed magnetic sensor system employed wireless connectivity, while it was cost-effective, and environmental-friendly. Through modelling of local magnetic field perturbations caused by a moving vehicle, the authors extracted the characteristics of magnetic waveforms for speed estimation.

HMC2003 three-axis magnetic sensor boards with HMC100x AMR sensing chips were used in the system presented in [19]. The authors built a roadside magnetic sensor system for vehicle detection.

The authors in [20] built a vehicle detection system as a wireless sensor network composed of a collection of sensor nodes put in the center of a lane with access point box used for the data collection. 

In [21] a vehicle detection state machine for magnetometer sensors for n-motorway lanes with multiple lane changes is presented. The proposed method can be used for suppressing the interference from a vehicle in an adjacent lane and lane changing.

QMC5883L modules, based on Honeywell HMC5883L AMR magnetometers, were used for the vehicle detection in [22] and could reliably detect the presence of a vehicle in a parking spot when placed under the front or rear axle of the vehicle.

LIS3MDL AMR magnetometers (STMicroelectronics) were used for the purposes of vehicle presence detection in [23]. The authors in this paper introduced a self-powered and autonomous sensing node equipped with supercapacitors, solar-based energy harvester and wake-up trigger prototype together with a Bluetooth Low-Energy (BLE) radio. The developed triggering system consumes only 150 nA.

Different AMR magnetometers were discussed in [24]—HMC1001, HMC1021 (both Honeywell), KMZ51 (NXP Semiconductors, Eindhoven, The Netherlands) and AFF755B (Sensitec GmbH, Wetzlar, Germany). According to the analysis, a new construction of sensors intended for vehicle detection experiments was presented.

The use of the magnetometers found in smartphones for the purposes of vehicle detection was presented in [25]. The system was used for the locating of street parking places by pedestrians.

From the analysis above is evident that the usage of AMR magnetometers for the purposes of road vehicle detection is very common, especially due to their relatively high accuracy which is in the contrast with low price and energy-efficiency which these sensors provide. After application of specific algorithms, these sensors can be easily used for almost full traffic surveillance, which involves vehicle classification, estimation of vehicle speed or traffic jam detection, eventually as a part of specific sensor systems [26]. After the implementation of appropriate software, magnetometer sensors could also provide a value-added contribution into the area of traffic surveillance, where other technologies take the role, as for example in sensing drunken drivers [27], as a part of driving assistance systems [28], for the edge traffic flow detection [29], as energy-efficient substitution of cameras for the detection and classification of road vehicles [30], or for the traffic abnormality detection [31] etc. 

Other low-cost technologies which could be used for vehicle detection were summarized in [32]. The authors summarized the progress in the area to help identify the sensing technologies with relatively high detection accuracy together with cost effectiveness and ease of installation. Special attention was paid to wireless battery-powered detectors of small dimensions that can be quickly and effortlessly installed alongside traffic lanes without any additional supporting structures. The methods of collecting traffic flow data were described in [33]. The authors tried to establish a low-cost wireless sensor network for providing data to advanced intelligent transportation system. The authors in [34] investigated a low-cost sensor network architecture for temporary installation on city streets as an alternative to commonly used rubber hoses. They presented the low-cost, low energy sensor together with the sensor location model. A case study with the installation of a set of proposed devices was presented to demonstrate its viability.

Different AMR magnetometers, as for example RM3100 (PNI Sensor Corporation), LIS3MDL (STMicroelectronics) or FXOS8700 sensors (NXP Semiconductors) have been used in some of the above articles. However, a direct comparison of the properties and performance of these sensors is lacking. The aim of this article is to compare these sensors and select the most suitable one for the implementation of the road traffic sensors that can be placed at the roadside.

## 3. Description of Sensors

The design of a road traffic sensor is a complex task. One of the most important steps is to select a suitable sensor. In [2,3] we tested 10 magnetometers in the laboratory. One of the main requirements when choosing magnetometers was their commercial availability. During testing, we focused on the size of the noise and the repeatability of measurements at different magnitudes of the magnetic field. For an analysis of magnetometers accuracy in terms of vehicle detection when placed by roadside, we selected the three best magnetometers (the RM3100, FXOS8700, and LIS3MDL or LSM303C). We also added the MLX90393 sensor, which is designed mainly for low-power devices. The next section lists the basic parameters of each selected sensor.

### 3.1. RM3100

This sensor manufactured by the PNI Sensor Corporation is exceptional in all respects. It is only tested sensor that does not use magneto-resistive technology, but rather is based on a LR oscillation circuit. It uses the property that the effective inductance of the coil is directly proportional to the magnitude of the magnetic field parallel to the orientation of the coil. The sensor uses three external coils connected to the control chip. Sampling frequency, scan sensitivity, and noise size are all related to the cycle count (CC) parameter, which can be set in the range 0-65535. Low CC values allow a high sampling rate to be used, but the resolution is lower. The situation is reversed for higher CC values. The manufacturer recommends using CC values in the range of 30–400. A different CC value can be set for each channel. The number of sensed channels affects the maximum sampling frequency. For CC = 50, the maximum sampling frequency is 1600/(number of channels). Magnetic field measurement range of the sensor is ±800 μT and the sensitivity is 75 LSB/μT for the default value of CC (200). This sensor is the only one of our selection to encode outputs using 24 bits. Other sensors are 16-bit. In addition to its advantages (high sampling frequency and high sensitivity), this sensor also has disadvantages: due to the need for external coils, the sensor is relatively large and also relatively expensive—especially compared to other sensors (see Table 1).

### 3.2. LSM303C and LIS3MDL 

These are representatives of the magnetometers manufactured by STMicroelectronics. In addition to the magnetometer, the LSM303C also includes an accelerometer, both of which are completely independent. The magnetometers of both sensors have very similar properties. In terms of management registers, they are almost identical. It can be said that the LIS3MDL is an improved version of the LSM303C magnetometer. The main difference is that the LIS3MDL supports the ranges ±4, ±8, ±12 and ±16 Gauss (±400 to ±1600 μT) and the "FAST" mode with support for a sampling frequency of 155 to 1000 Hz (depending on the required output quality). Interestingly, although the LSM303C has a single ±16 Gauss range according to the datasheet it is also possible to use (undocumented) ±4, ±8 and ±12 Gauss settings. The main advantage of these sensors is their higher sensitivity, which is for the range ±4 Gauss at the level of 6842 LSB/Gauss (or 68.42 LSB/μT). A certain limitation is the relatively low sampling frequency—for the LSM303C only 80 Hz and for the LIS3MDL 155 Hz (at maximum quality). The configuration of the sensors is relatively simple and consists in the selection of the range, sampling frequency and quality.

### 3.3. FXOS8700 

The FXOS8700 from NXP Semiconductors includes an accelerometer in addition to the magnetometer. The range of the sensor is quite large: ±1200 μT. This is associated with a lower sensitivity of 10 LSB/μT. The main advantage of the sensor is the high sampling frequency of 800 Hz, which does not depend on the number of measured channels. However, if we want to use an accelerometer at the same time as the magnetometer, the maximum sampling frequency will be halved, i.e., 400 Hz. The amount of noise can be affected by the over sample ratio (OSR) setting, the maximum size of which depends on the sampling frequency.

### 3.4. MLX90393 

This sensor from Melexis is designed for low-power applications. The sampling frequency of the sensor can be up to 700 Hz and depends on the over sample ratio setting and the way the output is filtered, with 8 different levels of filtration available. The sensitivity of the sensor is higher in the XY plane than in the Z axis and can reach a maximum of 6.211 LSB/μT for the X and Y axes and 3.406 LSB/μT for the Z axis. The sensitivity depends on the gain of the analog part and the method of selecting the 16 bits of output from the internal 19-bit analog-to-digital converter, with each channel having its own settings.

The main parameters of all the sensors, including average prices from most common shops dedicated to electronic components (we compare prices from the supplier mouser.com), are summarized in Table 1.

## 4. Hardware Platform

**Figure 1:**
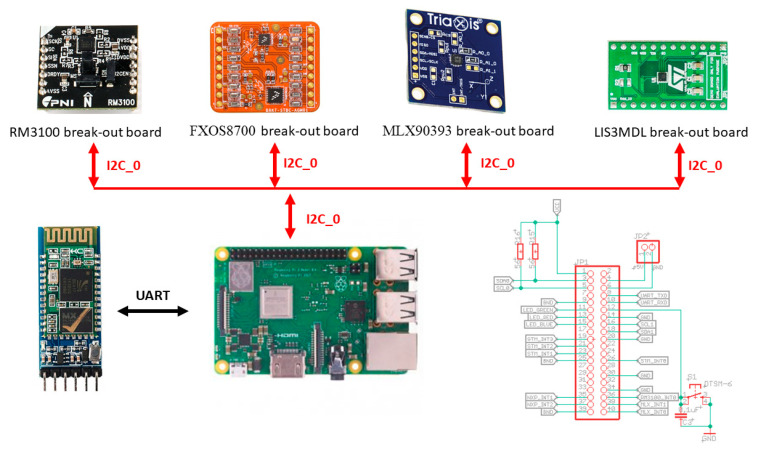
Block schematic of the developed measurement board.

**Figure 2:**
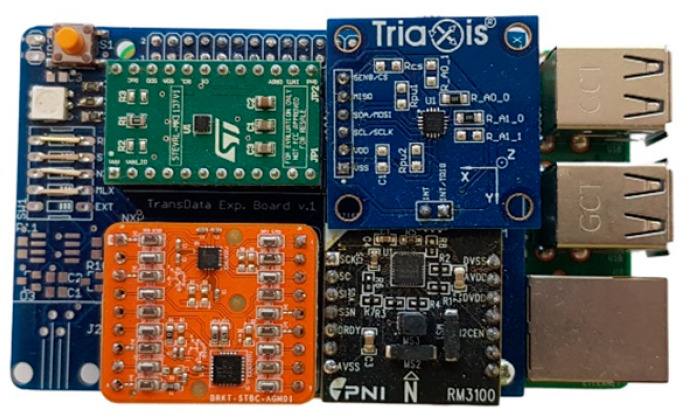
Completed measurement board with all magnetometer break-out boards installed in the top and Bluetooth module installed in the bottom (all attached to the developed shield).

To be able to test all sensors under the same conditions, particular break-out boards of the respective sensors were purchased. A break-out board makes it easy to use a single electrical component when adding the pins to the small package of magnetometer what allows connecting the board to a higher circuit. This makes it relatively easy to use. We decided to use RaspberryPi 3 Model B+ as the control board for setting up the sensor parameters, to start/end measurements and to store measured values in SD card. For this purpose, a special shield was designed and developed for RaspberryPi (see Figure 1 and Figure 2).

All selected sensors support serial peripheral interface (SPI) and inter-integrated circuit (I2C) communication interfaces. During our experiments, we used I2C in FAST mode (400 kHz). It can be seen also in the figures above, where all magnetometer break-out boards communicate with RaspberryPi via I2C interface. The Bluetooth module is connected through universal asynchronous receiver-transmitter (UART) interface. The measurement board also contains one indicating RGB LED diode and one development button for starting/stopping and resetting the measurements. External connectors allow connection of other possible sensors. Power supply was provided through standard 10 Ah Li-Ion Mini USB power bank, what was sufficient for the measurements lasting. Later on a developed Android application, which allowed an Android smartphone to connect to the measurement board, was used for the remote control of measurement and data download through Bluetooth. After all necessary testing procedures, the measurement board was further used for the sensors’ performance evaluation.

## 5. Laboratory Measurements

The real environment test of the sensors took place on the two-lane road I/64 in the First, we performed measurements in the laboratory to determine the effect of RaspberryPi (RPi) on the sensors. We tested two configurations: 1—placement of the expansion board directly on the RPi, 2—expansion board on the cable, approx. 40 cm from the RPi. For both configurations, two types of measurements were performed: scanning by each sensor separately and scanning by all sensors at once. During the measurements, the sensors were placed min. 2 m aside from sources of interference (computers, wiring). The length of each measurement was at least 20 seconds so that the number of readings was at least 2000 for each sensor. The measured values were stored in SD card located on the RPi and evaluated offline.

The settings of sensors were adjusted to maximize their sensitivity, while the sampling frequency was approximately 100 Hz. The main parameters of the sensors were set as follows (in parentheses is the actual sampling frequency—fs):

- RM3100: Cycle Count = 300 (fs = 104 Hz)
- LIS3MDL: range ±400 μT, Ultra-high-performance mode, FAST mode (fs = 157 Hz)
- FXOS8700: OSR = 8 (fs = 103 Hz)
- MLX90393: DIG_FILT = 2, OSR = 3, GAIN_SEL = 7 (fs = 96 Hz).

For each measurement, we calculated the variance, which reflects the magnitude of the noise. The measured noise is the sum of the sensor’s own noise and the noise induced from the environment. The results of measurements performed for each sensor separately are given in Table 2.

If we sort the sensors according to the size of the noise (from the smallest), the following order applies: RM3100, LIS3MDL, FXOS8700, MLX90393. The same order applies to all axes.

We expected that the measurement with sensors on the cable would contain the least noise, as the noise sources on the RPi will be more distant from the sensors. This has also been confirmed. With two minor exceptions, the amount of noise is lower for sensors located on a cable. However, the differences are relatively small, within a few percent. The only exception is the RM3100 sensor, which has very low intrinsic noise and at the same time high sensitivity. Therefore, it was most significantly affected by the noise generated by RPi. Nevertheless, even in the worst case (axis X), the amount of noise of the RM3100 is more than 3 times lower than that of the second-best sensor—LIS3MDL.

From a practical point of view, we are most interested in the X-axis, which is oriented towards the road if the sensors are located directly on the RPi and it is placed on the ground (parallel to the road). Differences between measurements of up to 19% were found in this axis.

In the second measurement, the magnetic field was sensed by all sensors at once. This means more current consumption and more frequent communication via the I2C bus. Therefore, we expected that the measured noise would be higher than in the previous measurement. The measurement results are shown in Table 3.

This measurement is more important to us because it corresponds to a situation of measuring vehicle records simultaneously with all sensors. The order of the sensors in terms of noise remained unchanged. When comparing the results with the previous measurement, we see that in almost all cases there was an increase in the measured noise. In the X-axis, which we are most interested in, the biggest percentage difference was with the FXOS8700 sensor—a change from 0.16698 (Table 2, board on cable) to 0.216764 (Table 3) represents an increase of almost 30%.

As in the first measurement, better results are obtained with the sensor board on the cable. The most notable exception is the RM3100 sensor, which has almost twice the noise in the X-axis compared to the placement of the sensors on the board. The X-axis was oriented parallel to the cable, and this apparently caused higher measured noise with this most sensitive sensor.

It should be noted that the comparison of sensors is not perfect. The differences in the measured noise are certainly also influenced by the mutual position of the sensor and RPi. However, this cannot be avoided, as we want to measure the deformation of the earth’s magnetic field by vehicles in real traffic with all sensors at once. The sensors are therefore necessarily differently oriented with respect to the individual noise sources.

After considering the results, we decided that due to the much simpler manipulation, we will use an expansion board located directly on the RPi during real measurements. Although this placement of expansion board increases the amount of measured noise (usually by a few percent) it should not significantly affect the performance of the sensors in detecting vehicles.

## 6. Real Traffic Measurements

The real environment test of the sensors took place on the two-lane road I/64 in the village of Porúbka (Slovakia) with daily traffic of 27,228 veh/day and rush-hour traffic of 2,051 veh/h (14% of trucks) measured by a Sierzega SR4 radar during a testing day. We performed two measurements at different distances of the sensors from the lane. In the first case, the sensor was placed on the outer edge of the road line and in the second case, it was at a distance of 0.5 m from the road line. In the previous measurements in [2], we evaluated the effect of sensor placement—we tested the placement directly on the ground and at a height of 30 cm above the ground. We found that the difference between the measured values is not significant. In these measurements, we decided to place the sensor directly on the ground. We wanted the sensor to be inconspicuous because we had found in the past that drivers tended to bypass the sensor when it was clearly visible and marked with a traffic cone.

**Figure 3:**
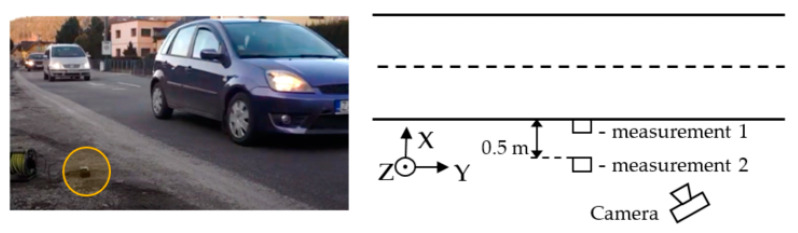
Sensor placement and view of the measuring set.

Measurements were taken between 16:45 and 17:30. We set the duration of both measurements so that the number of captured vehicles is approximately 100. For evaluation, passing vehicles were also recorded by a camera (with a frame rate of 24 fr/s). Figure 3 shows the location of the sensors during the measurements, the orientation of the axes relative to the road, and the location of the camera. The picture also shows a view of the measuring set.

During the first measurement, 88 vehicles were recorded, of which 74 were cars, nine vans, three buses and two trucks. In the second measurement, there were 94 vehicles, of which 86 were cars, seven vans and one truck. Based on the camera recording, each vehicle was assigned a type and time of passage (accurate to 1/24 s).

First, we evaluated the amount of noise from the measured data. According to the camera recording, we found a section in which no vehicles passed, and we calculated the variance. The length of the section was 20 s (comparable to noise measurements in the laboratory). The results are shown in Table 4.

Approximately the same level of noise was recorded in both measurements. The biggest differences are with the most sensitive sensor RM3100. If we compare a similar measurement performed in the laboratory (Table 3—joint measurement, placement of the expansion board on RPi), we find that in most cases slightly lower noise was measured in the exterior than in the interior. This result can be explained by stronger sources of interfering electromagnetic noise in the interior.

A much more important indicator of sensor quality is the achievable signal-to-noise ratio (SNR). Assuming a constant noise level, this ratio will depend on the distance of the sensor from the vehicle and the size of the passing vehicle. Based on the camera record, we found records of ten passenger cars and calculated the average SNR. The results are shown in Table 5. We used Equation (1) to calculate the SNR:

(1) $$SNR=10log\frac{var\left(CAR\right)}{var\left(NOISE\right)}$$

where *var*() represents the variance of a given signal and corresponds to its power.

This measurement fully showed the capabilities of the sensors, especially the sensitivity to relatively small changes in the Earth’s magnetic field caused by the passage of vehicles. The worst is again the MLX90393 sensor. This result was to be expected due to the low sensitivity of this sensor and at the same time its relatively high intrinsic noise. The RM3100 sensor achieved the best result, but the LIS3MDL provided good results too. This measurement also indicated the importance of placing the sensor as close as possible to the lane. Increasing the distance by 0.5 m resulted in a significant decrease in SNR (depending on the axis, the difference is from 3 to 8 dB for the RM3100 sensor). We assumed that the best results would be achieved in the X axis, which is oriented perpendicular to the road axis, and this was confirmed. An exception is the RM3100 sensor, which achieved the best SNR ratio in the Y axis (3.1–5.4 dB higher than in the X axis). It should be possible to use the values measured in both axes for the detection and eventual classification of vehicles. The Z axis at this sensor location contains the smallest information content.

**Figure 4:**
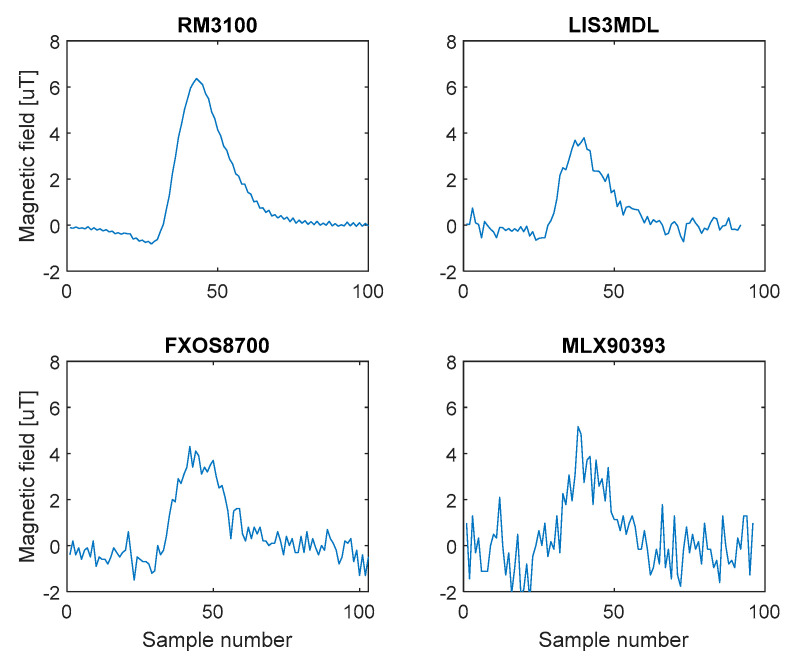
Record of Ford Focus combi in X axis.

**Figure 5:**
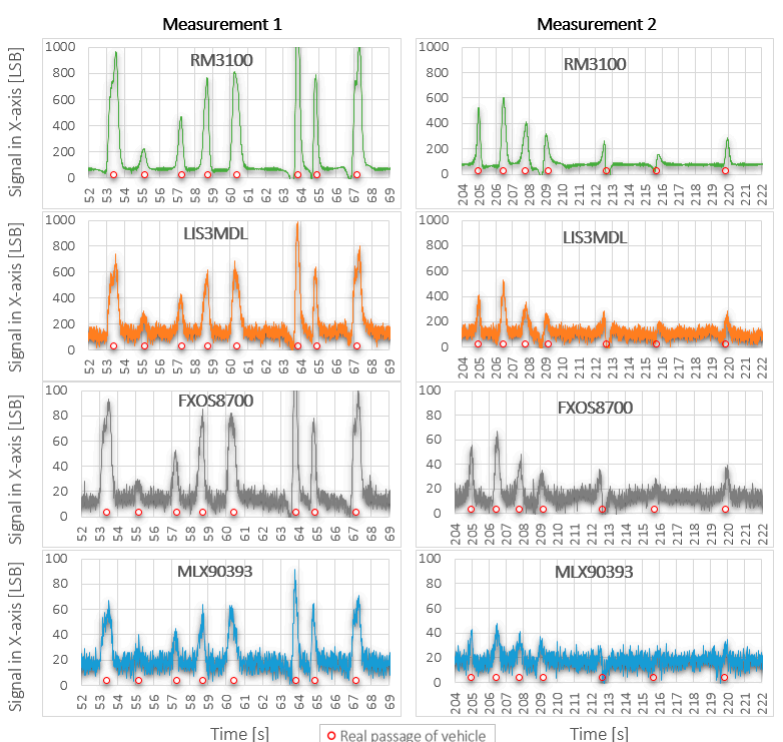
Comparison of recorded data by all sensors during both test measurements.

The different SNR level for the individual sensors is also visible in Figure 4, which shows the record of the Ford Focus combi with all sensors in the X-axis and in Figure 5, which shows a short part of recorded data by all sensors in the X-axis during both measurements. 

From the records in Figure 5, it is possible to observe a significant influence of the quality of the used sensor (sensitivity and intrinsic noise) and the distance of sensors from the road (measurement 1 versus measurement 2). Significantly higher deformation of the Earth’s magnetic field caused by real passage of vehicle was recorded by RM3100 and LIS3MDL sensors, and it is about 10 times higher in comparison with deformations obtained by FXOS8700 a MLX90393 sensors. The recorded deformation of magnetic field is reduced about half by moving all sensors by 0.5 m. Displayed results confirm that the RM3100 sensor is the best and the MLX90393 sensor is the worst of the tested magnetometers. The red dots in the figure mark the passing vehicle.

The most important test is to evaluate the success of vehicle detection by individual sensors. As part of pre-processing, it is necessary to remove noise and the DC component (offset) of the signal from the measured signals. The DC component of the signal depends on the size of the Earth’s magnetic field and is generally different at each place on Earth. A low-pass filter was used to suppress noise. After frequency analysis of the measured signals, we found that the useful signal contains frequency components up to about 2 Hz. Therefore, we chose the frequency of the low-pass filter at 2.5 Hz. The attenuation of the higher frequency components was 60 dB per decade. The DC component of the signal was calculated as the arithmetic mean of the values in that part of the signal where no vehicles passed. After removing the DC component, we found that the signals in some sensor axes still exhibited a certain offset that varied linearly over time. This linear offset was identified and removed. Only the RM3100 did not contain a linear offset in any axis. The worst in this respect was the LIS3MDL sensor, whose offset sometimes seemed to "float". Note that in real operation an algorithm for continuous offset detection and removal must be used.

We used two different approaches to detect vehicles—in the first case we evaluated only the signal in the most important X-axis and in the second case we also used data measured in other axes. In both cases, we intentionally used a simple detection algorithm to highlight the influence of the sensor properties (especially intrinsic noise and sensitivity) on the results obtained. A sophisticated detection algorithm could partially erase the differences between the individual sensors.

## 7. Detection Algorithm 

**Figure 6:**
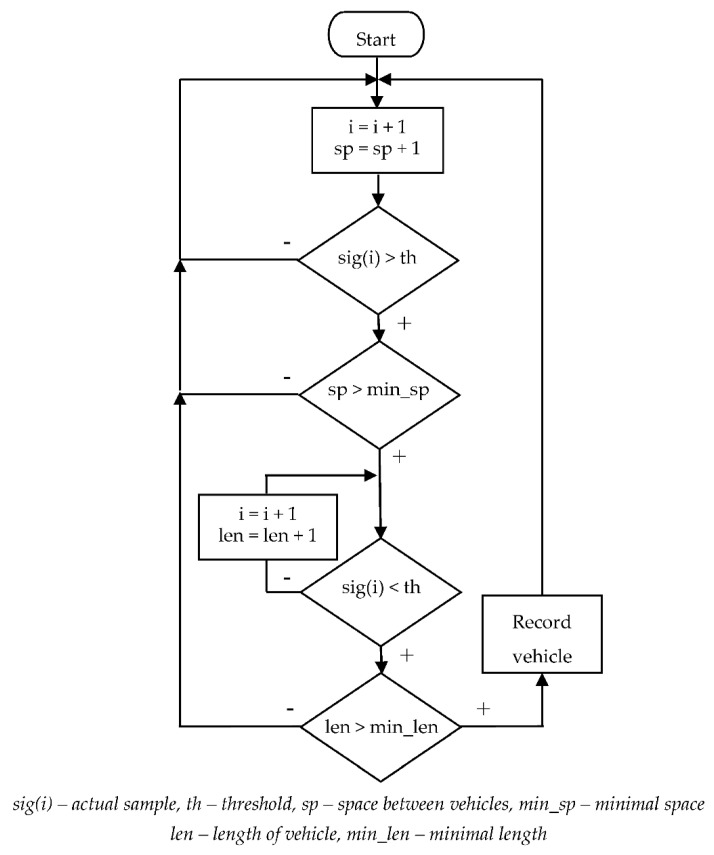
Detection algorithm principle.

The algorithm is based on the detection of the signal crossing over one threshold value. Therefore, the input signal must be non-negative (e.g., signal energy). When evaluating the signal, we considered the real properties of the vehicles and the traffic itself, namely the length of the vehicle and the gap between the vehicles. The length of vehicles is usually greater than 4 m. This, at a speed of 50 km/h (the municipal speed limit in Slovakia) and a sampling frequency of 100 Hz, represents approximately 29 samples per vehicle. The same goes for the gap between vehicles. We tested different settings for both parameters and selected the settings that achieved the best results. In one case the minimum vehicle length was 14 samples and the sample gap 21, in the other case the values 30 and 60 were used. The basic principle of the detection algorithm is shown in Figure 6. The algorithm assumes input signal with removed noise and zero offset.

**Figure 7:**
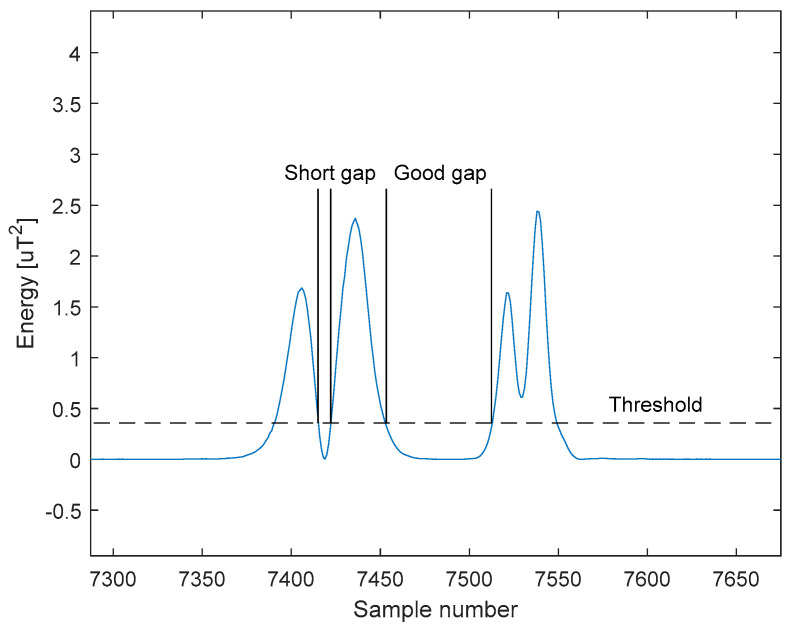
Example of gap length evaluation.

The algorithm successively compares all samples with the threshold. After exceeding the threshold, the length of the gap between the vehicles is checked. If the gap is sufficient, we assume that this is the beginning of the new vehicle record. If the gap is too small, we consider the record to be a continuation of the previous vehicle’s record. This part of the algorithm is not shown in Figure 6 for the sake of clarity. After detecting the vehicle, the algorithm waits for the signal to drop below a threshold value. The length of the vehicle is given by the number of samples that are above the threshold. The length must be greater than the given minimum value. In Figure 7 is an example of a gap that is too short and a gap that is long enough.

### 7.1. Application Scenario No.1—The Detection Based on the Single Signal

**Figure 8:**
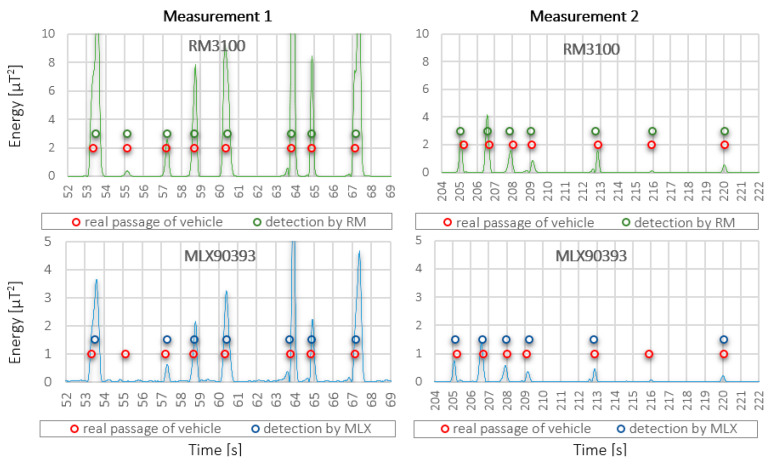
Comparison of the change of the input signal for detection at measurement 1 and measurement 2 for two selected sensors—RM3100 and MLX90393.

The energy of the most significant component of the signal (X-axis), which is proportional to the square of the signal, was used as the input signal for the detection algorithm. Example of the signal energy for the best sensor RM3100 and the worst sensor MLX90393 is shown in Figure 8. An important fact is that the input signal from the RM3100 sensor remains almost three times larger than the input signal from the MLX90393 due to its higher sensitivity and low level of intrinsic noise. This has a direct effect on the success of the detection, especially in cases of very low response from the passing vehicle (e.g., if the vehicle passed in the measuring profile at a greater distance from the sensors). This case can be observed from the detection results of both sensors, for both measurements in the graphs in Figure 8, where the results of detection by a given sensor are displayed (green and blue dots), as well as the actual vehicle crossings determined from the camera recording (real passage of vehicle, red dots). The method of evaluation and the results of the detection itself for all sensors are given below for both measurements.

**Figure 9:**
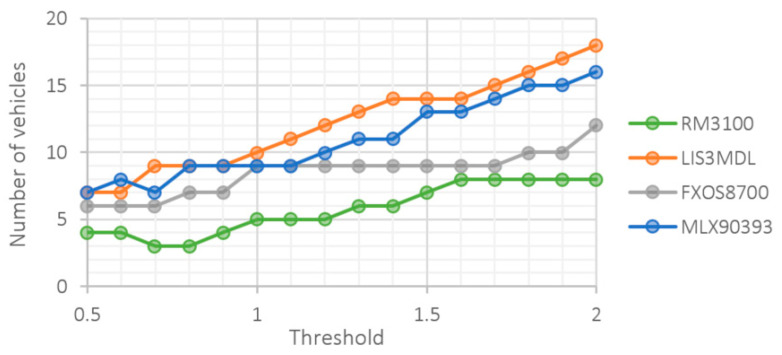
Number of undetected vehicles at different threshold level.

The vehicle detection results based on the input signal are highly dependent on the specific value of the threshold. When choosing it, a compromise must be made between the number of undetected vehicles and the number of false positives. The lower the threshold, the more vehicles we can detect. However, at the same time, the algorithm is more prone to exceeding the threshold value due to noise or offset change, which is a false positive. Since we do not know in advance which threshold is the best, we performed detection for different thresholds. Figure 9 shows the dependence of the number of undetected vehicles on the size of the threshold value for all sensors.

The number of undetected vehicles is expected to increase with increasing threshold size. This does not only apply to very low threshold levels, when several records are “merged” into one, especially for sensors with higher intrinsic noise. The best result was achieved by the RM3100 sensor. It is followed by FXOS8700 and at the end LIS3MDL with MLX90393 were placed. Given the proclaimed, relatively high sensitivity of the LIS3MDL sensor and the amount of noise and SNR compared to the FXOS8700 sensor, this result is quite surprising.

**Figure 10:**
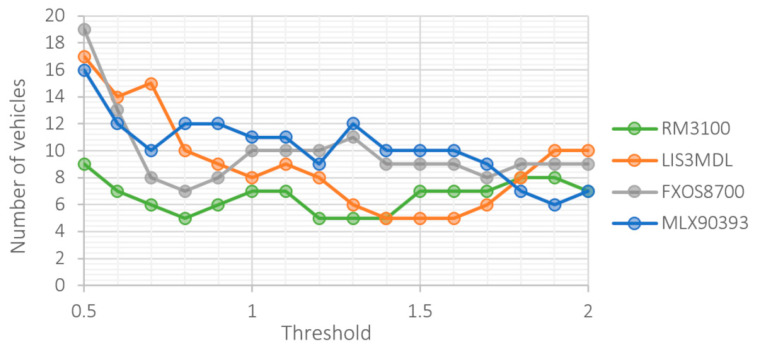
Number of false positives at different threshold level.

Figure 10 shows the dependence of the number of false positives on the size of the threshold value. Due to the higher noise of the LIS3MDL, FXOS8700 and MLX90393 sensors, frequent false detections occur at low thresholds.

**Figure 11:**
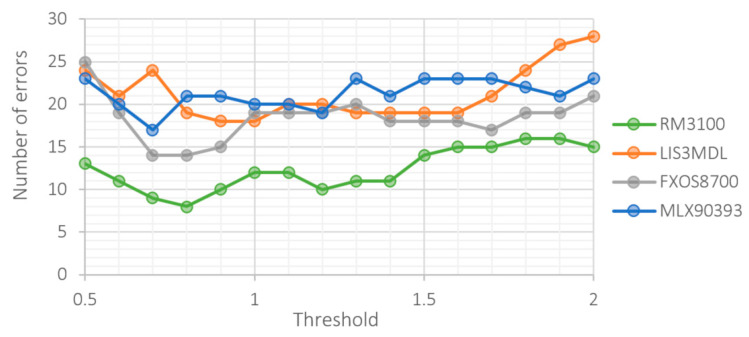
Total number of errors at different threshold level.

Figure 11 shows the total number of errors—the sum of undetected vehicles and false positives.

All three last images were obtained for parameter settings 14/21 (minimum vehicle length and minimum gap). For the 30/60 setting, the number of undetected vehicles increases, and the number of false positives decreases. In both cases, the reason is the tightening of requirements.

The best sensor is the RM3100, which can keep false positives low over a wide range of threshold levels. This is due to the very low noise of this sensor. Upon closer examination of the reasons of false positives for the RM3100 sensor, we found that they were all caused by the passage of trucks in the opposite direction. It is a “tax” for its high sensitivity.

Comparing Figure 9 and Figure 10, we see that sensors achieve the best results at different threshold levels. A trade-off needs to be made between the sensor’s ability to detect vehicles and the number of false positives. In this respect, the RM3100 sensor is again the best. The detection ability of this sensor is not as sensitive to the level of the threshold as it is for other sensors. This allows to choose a threshold value that is optimal in both respects.

Table 6 shows the optimal threshold level for each sensor together with the achieved results. We determined the optimal threshold level by taking the minimum of the sum of undetected vehicles and false positives.

For all sensors, the best results were obtained for detection parameters 30/60. In this case, there are more undetected vehicles and a low number of false positives. If we require a lower number of undetected vehicles, it is better to use the 14/21 setting.

The results of the second measurement, with the sensor located at a distance of 0.5 m from the lane, are shown in Table 7.

Despite the greater distance of the sensor from the road, the sensors achieved better results than in the first measurement. This result may seem paradoxical, but it cannot be generalized. The change in magnetic field caused by large vehicles in the opposite direction can be detected by sensors and usually manifests as false positive but can also cause the records of several vehicles to be combined into one. Of course, the composition of the vehicles in both directions was not the same in both measurements, so different number of false positives and/or combined records were generated. The influence of oncoming vehicles can be reduced by using several sensors placed parallel to the road. Exploring this option is beyond the objectives of this article.

### 7.2. Application Scenario No.2—The Detection Based on the Multiple Signals

In this section, we focused on identifying opportunities to improve detection through the use of signals measured in different axes. We examined all combinations of signals measured in two axes, while we used the following relations to combine two signals into one: A^2^ + B^2^, (A + B)^2^, abs(A) + abs(B) and arctan(A/B) (according to [19]). For the sensors RM3100, FXOS8700 and MLX90393, the best results were obtained with the sum of the signal energies in the X and Y axes, i.e. X^2^ + Y^2^. Only the LIS3MDL sensor shows the best results for the combined signal X^2^ + Z^2^. Table 8 shows the best results obtained for the first measurement.

The use of values from two axes led to significantly better, or at least the same, results as when using one axis (comparison with Table 6). This time, better results were obtained for detection parameters 14/21. This is because adding the energies of the two signals increases the width of the vehicle’s recording and decreases the gap between vehicles. If a longer gap is required (60 vs. 21), the vehicle records are removed more frequently. Adding the two signals also requires increasing the size of the threshold. The LIS3MDL sensor is the only one to achieve the best results for the combination of X and Z axes. For other sensors, the combination of X and Y axes is the best.

We evaluated the second measurement in the same way. For all sensors, the best results were obtained for the combined signal X^2^ + Y^2^. Table 9 shows the achieved results.

Unlike the first measurement, the results have now only been improved for the MLX90393 sensor. The results of the RM3100 did not improve and the results of the other two sensors were even worse. The percentage of deterioration looks significant, but in reality, it was only an increase in the number of errors by one and two (respectively LIS3MDL and FXOS8700).

## 8. Summary of Results

To determine the real properties of the sensors (RM3100, LIS3MDL, FXOS8700, and MLX90393), measurements were performed in the laboratory and under real conditions. Measurements in the laboratory were aimed at determining the amount of noise that can be expected when measuring with the sensors. The magnitude of the measured noise depends on the sensor’s own noise, the noise generated by the sensing device and the ambient noise. We minimized the ambient noise mainly by increasing the distance of the sensors from the main sources of interference (computers and electrical wiring). The effect of the sensing device itself, which is based on the RPi, was tested in two ways—by placing the expansion board with sensors directly on the RPi and by distancing the expansion board from the RPi with a cable. 

The first measurement was performed for each sensor separately. The measurement results showed that the magnitude of the measured noise is lower when placing the expansion board on the cable. The differences of noise level were small, within a few percent. Significant differences were recorded only for the RM3100 sensor, which is the most sensitive of all tested sensors.

In the second measurement, the noise was measured simultaneously by all sensors. Simultaneous sensing by all sensors causes increased current consumption and also more frequent communication via the I2C bus, which is reflected in an increase in the measured noise. The largest percentage difference compared to the first measurement was recorded for the FXOS8700 sensor (almost 30% for the X-axis). As in the first measurement, lower noise was measured when the expansion board was placed on the cable. The exception was the RM3100 sensor, which recorded in the X-axis almost twice as much noise on the cable as on the board. This anomaly was caused by the orientation of the cable parallel to the X-axis of the sensor and its high sensitivity.

In both measurements in the laboratory, the order of the sensors according to the amount of noise was as follows (sorted from the smallest noise to the largest): RM3100, LIS3MDL, FXOS8700 and MLX90393. 

Despite the higher measured noise value (usually by a few percent), we decided to use sensors placed directly on the expansion board in further measurements. The decisive factor was the much simpler handling of the sensing device.

The performance of sensors in vehicle detection was tested in two measurements performed on a local two-lane road. In the first case, the sensor was placed on the outer edge of the road line and in the second case, it was at a distance of 0.5 m from the road line. First, we evaluated the amount of noise, which was slightly lower than in the laboratory measurements. This is due to the smaller number of sources of interfering electromagnetic noise in the exterior.

The ability of the sensors to detect the vehicle is largely dependent on the magnitude of the signal-to-noise ratio, i.e., the SNR. Therefore, for both measurements, we calculated the average SNR value from the records of ten passenger cars. This measurement showed how important it is to place the sensor as close to the lane as possible. Increasing the distance by 0.5 m results in a significant decrease in SNR (depending on the axis, the difference is from 3 to 8 dB for the RM3100 sensor). The highest SNR values were recorded by the RM3100 sensor. This test also showed that the highest SNR is achieved in the X and Y axes.

In order to compare the performance of sensors in vehicle detection, an algorithm based on the evaluation of the measured signal energy transition over a threshold value was used. The X-axis signal was used for evaluation. During preprocessing the DC component was removed from the measured signal and the noise was suppressed by a low-pass filter. Only the RM3100 sensor has not any offset. The worst in terms of preprocessing is the LIS3MDL sensor, whose offset seems to “float”. For the other sensors, the offset was a linear function of time.

The performance of the detection algorithm depends on the level of the threshold. Therefore, we evaluated each sensor for different threshold levels. In addition to the size of the threshold, we tested various settings for the minimum length of the vehicle and the minimum gap between vehicles. When comparing sensor performance, we used the best settings found for each sensor separately. We evaluated the number of undetected vehicles and the number of false positives. The RM3100 sensor achieved the best results in both measurements. The high sensitivity of the RM3100 sensor causes a low number of undetected vehicles (3.4% and 1.1% in the first and second measurements) but on the other hand a higher number of false positives, which were caused by the detection of vehicles moving in the far lane. This sensor is also the least sensitive to the specified threshold level. The increased sensitivity of other sensors to the size of the threshold in practice means higher demands on the processing of input data, as well as on the detection algorithm itself. From this point of view, the RM3100 sensor offers a certain stability during detection, even in the most unfavorable detection conditions (e.g., greater distance of the vehicle from the sensor when passing through the measuring profile, lower volume of ferromagnetic parts in the vehicle, etc.).

We also tested the possibility of improving detection by using signals measured in multiple axes. The results obtained are ambiguous. In the first measurement, the detection was improved for three sensors, but in the second measurement only for one sensor and for the other two, the results were even worsened. From this point of view, the RM3100 sensor was again the best, which achieved an improvement of 45% in the first measurement and in the second there was neither improvement nor deterioration of the results.

A summary of the achieved results is given in Table 10 in the form of the score. We determined the order of individual sensors for each evaluated area. When evaluating the success of the detection, we took into account the average of the results of both measurements.

For the overall evaluation, we considered only the first six criteria. Size, price, and energy consumption are not very important for the development of a sensor for short-term traffic survey.

The RM3100 sensor was the best in all important areas. Its high sensitivity and low noise allow the best detection of vehicles, even at a greater distance of the sensor from the road. The disadvantage of high sensitivity is the higher susceptibility to errors caused by the passage of vehicles in the opposite direction, which, however, can be eliminated by a more sophisticated detection algorithm. In second place is FXOS8700, closely followed by LIS3MDL. The MLX90393 sensor was placed last, which is mainly due to its higher noise combined with lower sensitivity. However, even its results are not unusable, especially when measuring near the lane. At longer distances (more than 1.0 m), there is a significant increase in the risk that the response from a passing vehicle will be literally "buried" in the sensor’s own noise.

## 9. Conclusions

Our goal was to compare selected magnetometers in terms of the possibility of their use to detect vehicles when placing the sensor next to the road. An expansion board for the RaspberryPi was designed for simultaneous measurement with all sensors. With the created sensing device, initial tests were performed under laboratory conditions, while we evaluated the amount of noise measured by the sensors at different configurations of the measuring device.

The most important were the measurements made on the road with real traffic. We performed two measurements at different distances from the road. We evaluated the measured signals using an algorithm for vehicle detection based on signal energy. We found the best settings of the detection algorithm parameters for each sensor. We also investigated the possibility of vehicle detection based on the evaluation of signals from several axes of the magnetometer.

The order of the sensors was as follows: RM3100, FXOS8700, LIS3MDL and MLX90393. The RM3100 sensor achieved the best results in all tests. This sensor has the highest sensitivity and the lowest intrinsic noise. These features allow the sensor to be placed at greater distances from the road, which is very important due to the possibility of placing automatic traffic counters during traffic surveys. A good feature of the RM3100 is also the possibility of increasing its sensitivity at lower sampling frequency and/or a smaller number of scanned axes. The main disadvantages of the RM3100 include its larger dimensions and by far the highest price.

These results are only the first stage in designing a suitable sensor for the magnetic traffic counter to be placed at the roadside.

The analysis presented in the paper will serve to the future research when the special sensor node for traffic surveillance will be designed. With respect to the paper findings, magnetometer RM3100 will be used as the main sensing unit for this node. According to the SoA analysis in Section 2, it is expected that magnetometer pair spaced by the exact distance of min. 10 cm will provide sufficient performance for accurate road vehicle detection even in if vehicle will pass during traffic jam. For detection purposes, bandpass filter together with local maxima detection on correlated signals from both magnetometers should be applied. Vehicle classification will be then based on the convolutional neural network applied to the recorded detections. For this purpose, the building of robust dataset belongs to one of the most important tasks of our future research.

## Figures and Tables

**Table 1:** Important parameters of sensors.

Sensor	Range [μT]	Sensitivity [LSB/μT]	Sampling Frequency [Hz]	Unite Price [€]
RM3100	±800	>100 ^1^	1600/n ^1^	12.75
LIS3MDL/LSM303C	±400	68.42	80/155	1.37
FXOS8700	±1200	10	800	3.41
MLX90393	±4800	6.21	717	1.65

^1^ Depends on Cycle Count setting.

**Table 2:** Variance of noise for individual measurements [μT^2^].

Axis X	RM3100	LIS3MDL	FXOS8700	MLX90393
Board on RPi	0.01885	0.061643	0.187634	0.863626
Board on cable	0.015285	0.062874	0.16698	0.845773
Difference [%]	−18.9	+2.0	−11.0	−2.1
**Axis Y**	**RM3100**	**LIS3MDL**	**FXOS8700**	**MLX90393**
Board on RPi	0.00279	0.05997	0.158713	0.729022
Board on cable	0.002567	0.05807	0.152333	0.736557
Difference [%]	−8.0	−3.2	−4.0	+1.0
**Axis Z**	**RM3100**	**LIS3MDL**	**FXOS8700**	**MLX90393**
Board on RPi	0.004791	0.12119	0.374178	1.942289
Board on cable	0.003306	0.1206965	0.369923	1.768549
Difference [%]	−31.0	−0.4	−1.1	−8.9

**Table 3:** Variance of noise for joint measurements [μT^2^].

Axis X	RM3100	LIS3MDL	FXOS8700	MLX90393
Board on RPi	0.012109	0.071062	0.241074	0.907603
Board on cable	0.023884	0.065095	0.216764	0.890775
Difference [%]	+97.2	−8.4	−10.1	−1.8
**Axis Y**	**RM3100**	**LIS3MDL**	**FXOS8700**	**MLX90393**
Board on RPi	0.002546	0.074101	0.195486	0.721353
Board on cable	0.002911	0.059963	0.160871	0.762524
Difference [%]	+14.3	−19.1	−17.7	+ 5.7
**Axis Z**	**RM3100**	**LIS3MDL**	**FXOS8700**	**MLX90393**
Board on RPi	0.005866	0.185112	0.438575	1.896881
Board on cable	0.003518	0.126429	0.447737	1.861972
Difference [%]	−40.0	−31.7	+ 2.1	−1.8

**Table 4:** Amount of noise in real conditions [μT^2^].

Sensor	Measurement 1			Measurement 2		
	X	Y	Z	X	Y	Z
RM3100	0.0028	0.0011	0.0104	0.0089	0.0007	0.0228
LIS3MDL	0.0899	0.0650	0.0935	0.0720	0.0692	0.1019
FXOS8700	0.1939	0.1740	0.4627	0.1823	0.1482	0.4200
MLX90393	0.6430	0.5899	1.7458	0.6419	0.6464	1.6755

**Table 5:** Mean SNR [dB] of a passenger car.

Sensor	Measurement 1			Measurement 2		
	X	Y	Z	X	Y	Z
RM3100	30.0	33.1	14.7	22.5	27.9	11.7
LIS3MDL	11.7	10.2	5.9	8.4	6.8	2.3
FXOS8700	10.9	9.1	3.2	6.9	5.0	1.2
MLX90393	6.4	4.2	1.1	3.5	1.6	0.3

**Table 6:** Optimal threshold level and detection results for measurement 1.

Detection Parameters	Observed Characteristics	RM3100	LIS3MDL	FXOS8700	MLX90393
14/21	Threshold	0.8	0.9	0.8	0.7
	Undetected	3 (3.4%)	9 (10.2%)	7 (8.0%)	7 (8.0%)
	False	8 (9.1%)	9 (10.2%)	7 (8.0%)	10 (11.4%)
	Errors	11	18	14	17
30/60	Threshold	1.2	0.9	0.9	0.7
	Undetected	8 (9.1%)	12 (13.6%)	10 (11.4%)	11 (12.5%)
	False	3 (3.4%)	2 (2.3%)	3 (3.4%)	2 (2.3%)
	Errors	11	14	13	13

**Table 7:** Optimal threshold level and detection results for measurement 2.

Detection Parameters	Observed Characteristics	RM3100	LIS3MDL	FXOS8700	MLX90393
14/21	Threshold	0.5	0.3	0.8	0.8
	Undetected	1 (1.1%)	1 (1.1%)	5 (5.3%)	10 (10.6%)
	False	5 (5.3%)	9 (9.6%)	3 (3.2%)	3 (3.2%)
	Errors	6	10	8	13
30/60	Threshold	1.4	0.3	0.8	0.8
	Undetected	7 (7.4%)	9 (9.6%)	5 (5.3%)	11 (11.7%)
	False	2 (2.1%)	9 (9.6%)	2 (2.1%)	2 (2.1%)
	Errors	9	18	7	13

**Table 8:** Detection parameters and results for measurement 1.

Observed Characteristics	RM3100	LIS3MDL	FXOS8700	MLX90393
Detection parameters	14/21	14/21	14/21	14/21
Axes	X^2^ + Y^2^	X^2^ + Z^2^	X^2^ + Y^2^	X^2^ + Y^2^
Threshold	0.9	1.4	1.3	0.9
Undetected	3 (3.4%)	5 (5.7%)	6 (6.8%)	8 (9.1%)
False	3 (3.4%)	3 (3.4%)	4 (4.5%)	5 (5.7%)
Errors	6	8	10	13
Change in%	45	33	23	0

**Table 9:** Detection parameters and results for measurement 2.

Observed Characteristics	RM3100	LIS3MDL	FXOS8700	MLX90393
Detection parameters	14/21	14/21	14/21	14/21
Axes	X^2^ + Y^2^	X^2^ + Y^2^	X^2^ + Y^2^	X^2^ + Y^2^
Threshold	0.6	1.0	1.2	0.9
Undetected	1 (1.1%)	1 (1.1%)	7 (7.4%)	8 (8.5%)
False	5 (5.3%)	10 (10.6%)	2 (2.1%)	2 (2.1%)
Errors	6	11	9	10
Change in%	0	−10	−28	23

**Table 10:** Summary of the achieved results.

Parameter	RM3100	LIS3MDL	FXOS8700	MLX90393
Sensitivity	1	2–3	2–3	4
Noise level	1	2	3	4
SNR	1	2	3	4
Offset	1	4	2	3
Detection—one axis	1	3–4	2	3–4
Detection—two axes	1	3	2	4
Size	4	1	3	2
Price	4	1	3	2
Energy consumption ^1^	4	3	1	2

^1^ Based on datasheet values.

## Data Availability

The data presented in this study are available on request from the corresponding author.
